# Widespread cortical and subcortical gray matter loss and an increase of globus pallidus volume in treatment-resistant schizophrenia

**DOI:** 10.1192/j.eurpsy.2024.1281

**Published:** 2024-08-27

**Authors:** A. Dudina, D. Tikhonov, V. Kaleda, I. Lebedeva

**Affiliations:** ^1^Laboratory of Neuroimaging and Multimodal Analysis; ^2^Department of Youth Psychiatry, Mental Health Research Center, Moscow, Russian Federation

## Abstract

**Introduction:**

It is still being discussed whether treatment-resistant schizophrenia (TRS) is a biological subtype which differs from non-treatment-resistant schizophrenia or is it a more severe condition that affects brain worse than non-treatment-resistant schizophrenia. However, there are few and heterogeneous studies and the etiology of TRS remains quite unclear.

**Objectives:**

This study aimed to explore cortical and subcortical morphometric characteristics in TRS patients and its associations with the clinical features. The pilot stage comprises the comparison to the mentally healthy subjects.

**Methods:**

21 right-handed male patients (mean age 28.99 ± 8.08 years) fulfilling TRS criteria and 21 matched healthy controls (mean age 29.35 ± 7.41 years) underwent T1-weighted structural MRI at 3T Philips scanner and clinical examination. Images were processed using FreeSurfer 7.1.1. Cortical thickness and area, volumes of subcortical structures and separately volumes of the amygdala nuclei and hippocampal subregions were compared between groups. The morphometry data, PANSS (Positive and Negative Syndrome Scale), CDSS (Calgary Depression Scale for Schizophrenia) and daily chlorpromazine equivalent doses of antipsychotics were included in correlational analysis. Results were considered significant if they retained significance after correction for multiple comparisons.

**Results:**

Compared to healthy controls, TRS patients showed decreased gray matter thickness in frontal, temporal, parietal, occipital, cingulate and insular regions (Figure 1). The temporal lobe showed the most prominent thinning of the cortex. The volumes of the amygdala, hippocampus (Figure 2) and nucleus accumbens, a number of amygdala nuclei and hippocampal subregions bilaterally were also decreased in TRS patients. The volume of the right globus pallidus, on the contrary, was increased (Figure 2). No correlations between altered cortical thickness, PANSS (positive, negative, general psychopathology scales and total score), CDSS and chlorpromazine equivalent doses of antipsychotics were found.

**Image:**

**Figure:**
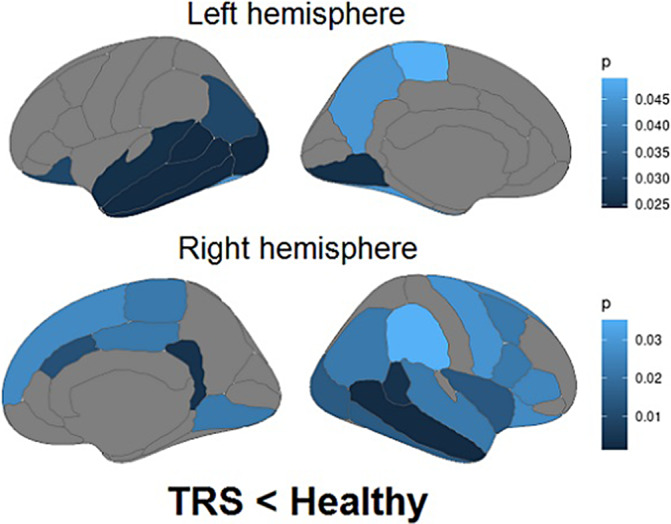

**Image 2:**

**Figure:**
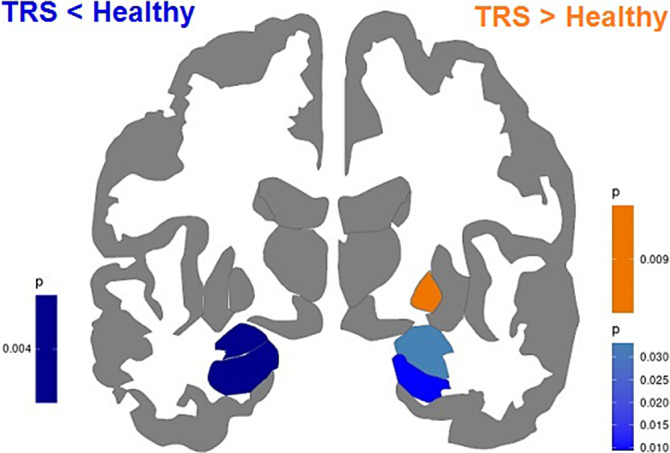

**Conclusions:**

The widespread gray matter cortical and subcortical loss in TRS finds confirmation in the literature. The increased globus pallidus volume is an unexpected and intriguing result. Other studies demonstrated conflicting results on that point. Some studies reported possible therapy influence, others suggested possible associations with symptoms. We did not find any correlations with psychometric or therapy characteristics. It is possible that there are non-linear relationships or relationships that exist only at a certain stage of the disease. As for therapy, patients took individual medication, consisting of various antipsychotics and drugs from other pharmacological groups, and such heterogeneity could affect the results of the study. Further research is going to be carried out.

**Disclosure of Interest:**

None Declared

